# SepsEast Registry indicates high mortality associated with COVID-19 caused acute respiratory failure in Central-Eastern European intensive care units

**DOI:** 10.1038/s41598-022-18991-2

**Published:** 2022-09-01

**Authors:** Jan Benes, Miłosz Jankowski, Konstanty Szułdrzynski, Roman Zahorec, Mitja Lainscak, Zoltán Ruszkai, Matej Podbregar, Jan Zatloukal, Jakub Kletecka, Krzysztof Kusza, Jakub Szrama, Estera Ramic, Katarina Galkova, Stefan Krbila, Josef Valky, Jaka Ivanic, Marko Kurnik, Angéla Mikó, Tamás Kiss, Barbara Hetényi, Peter Hegyi, Alan Sustic, Zsolt Molnar

**Affiliations:** 1 Department of Anesthesiology and Intensive Care Medicine, Faculty of Medicine in Pilsen, Charles University, Pilsen, Czech Republic; 2 Department of Anesthesiology and Intensive Care Medicine, University Hospital Pilsen, Pilsen, Czech Republic; 3 Biomedical Centre, Faculty of Medicine in Pilsen, Charles University, Pilsen, Czech Republic; 4 Department of Anesthesiology and Intensive Therapy, Central Clinical Hospital of the Ministry of Interior and Administration, Warsaw, Poland; 5 Jagiellonian University Medical College, Krakow, Poland; 6 Anesthesiology and Intensive Medicine, Medical School, Comenius University, Bratislava, Slovakia; 7 Division of Cardiology, General Hospital Murska Sobota, Murska Sobota, Slovenia; 8 Faculty of Medicine, University of Ljubljana, Ljubljana, Slovenia; 9 Department of Anesthesiology and Intensive Therapy, Flór Ferenc Hospital County Pest, Kistarcsa, Hungary; 10 Department for Internal Care Medicine, General Hospital Celje, Celje, Slovenia; 11 Department of Anesthesiology and Intensive Therapy and Pain Management, Poznan University of Medical Sciences, Poznan, Poland; 12 Department of Anesthesiology, Reanimatology, Intensive Care and Emergency Medicine, Faculty of Medicine, University of Rijeka, Rijeka, Croatia; 13 Department of Anaesthesiology and Intensive Care, Faculty Hospital, Nitra, Slovakia; 14 Department of Anaesthesia and Intensive Therapy, University Hospital Nové Zámky, Nové Zamky, Slovakia; 15 Department Anesthesiology and Intensive Therapy, University Hospital Banska Bystrica, Banska Bystrica, Slovakia; 16 Department of Ananesthesiology and Perioperative Medicine, General Hospital Murska Sobota, Murska Sobota, Slovenia; 17 Department of Anesthesiology and Intensive Therapy, School of Medicine, University of Pécs, Pécs, Hungary; 18 Institute for Translational Medicine, Medical School, Szentágothai Research Centre, University of Pécs, Pécs, Hungary; 19 Centre for Translational Medicine, Semmelweis University, Budapest, Hungary; 20 Division for Pancreatic Disorders, Heart and Vascular Center, Semmelweis University, Budapest, Hungary; 21 Department of Clinical Medical Science II, Faculty of Health Studies, University of Rijeka, Rijeka, Croatia; 22 Department of Anaesthesiology and Intensive Therapy, Semmelweis University, Budapest, Hungary

**Keywords:** Medical research, Outcomes research

## Abstract

The coronavirus disease (COVID-19) pandemic caused unprecedented research activity all around the world but publications from Central-Eastern European countries remain scarce. Therefore, our aim was to characterise the features of the pandemic in the intensive care units (ICUs) among members of the SepsEast (Central-Eastern European Sepsis Forum) initiative. We conducted a retrospective, international, multicentre study between March 2020 and February 2021. All adult patients admitted to the ICU with pneumonia caused by COVID-19 were enrolled. Data on baseline and treatment characteristics, organ support and mortality were collected. Eleven centres from six countries provided data from 2139 patients. Patient characteristics were: median 68, [IQR 60–75] years of age; males: 67%; body mass index: 30.1 [27.0–34.7]; and 88% comorbidities. Overall mortality was 55%, which increased from 2020 to 2021 (p = 0.004). The major causes of death were respiratory (37%), cardiovascular (26%) and sepsis with multiorgan failure (21%). 1061 patients received invasive mechanical ventilation (mortality: 66%) without extracorporeal membrane oxygenation (n = 54). The rest of the patients received non-invasive ventilation (n = 129), high flow nasal oxygen (n = 317), conventional oxygen therapy (n = 122), as the highest level of ventilatory support, with mortality of 50%, 39% and 22%, respectively. This is the largest COVID-19 dataset from Central-Eastern European ICUs to date. The high mortality observed especially in those receiving invasive mechanical ventilation renders the need of establishing national–international ICU registries and audits in the region that could provide high quality, transparent data, not only during the pandemic, but also on a regular basis.

## Introduction

The coronavirus disease (COVID-19) caused unprecedented burden to the whole community and exhausted healthcare systems worldwide^1,2^. From the beginning it became clear that the highest risk of dying is among those patients who develop severe respiratory failure and require invasive mechanical ventilation in the intensive care units (ICUs)^3–5^. The first wave spared most of the Central-Eastern European countries from the former socialist block. However, the death toll, as defined by deaths per million inhabitants during the second and especially the third waves within our region exceeded those reported from the majority of Western Europe^6^. Despite the unification of Europe almost 20 years ago, the plausible differences in health care systems and clinical research activities between Western European and Central-Eastern European countries remain. These are well acknowledged in general, but rarely analysed or reported in scientific papers^7^. The existence of these differences is further supported by a recent study which evaluated and ranked excessive deaths in 29 high income countries associated with the COVID-19 pandemic in 2020, in which several Central-Eastern European countries were listed among the highest^8^.

After the first COVID-19 related reports in 2020, the SepsEast community, which is a voluntary initiative of Central-Eastern European intensivists founded in 2012 and covering the whole region^7^, tried to react to the challenges of the COVID-19 pandemic, and started research and various educational activities in the region^9^. One such activity was the development of the SepsEAst Registry to define the CHaracteristics in COronaVIrus Disease 2019 (SEARCH-COVID-19). The aim of the SEARCH-COVID-19 study was to collect structured data from ICUs within the SepsEast community during the pandemic.

## Methods

### Ethics

The original study protocol was first approved by the Hungarian National Research and Ethics Committee (IV/3971-3/2020/EKU) in May 2020 (Nemzeti Népegészségügyi Központ IV/3971-3/2020/EKU, 20.05.2020), and then in all other participating centres. However, this study did not include any patients. Therefore, in 2021 we submitted the approval of retrospective data collection Ethical Committee University Hospital Pilsen and Faculty of Medicine in Pilsen, Charles University, reference number: 198/2021, and then in all other participating centres (list of the local ethics committees is detailed in the “Ethics approval and consent to participate” section). Because of its retrospective nature and handling of anonymised data, no patient approvals or consents were deemed necessary and were waived by the various local ethics committees.

### Study design and setting

Originally the study was designed to be a prospective registry. However, due to the overwhelming workload and staff shortages during the actual waves, it was impossible to prospectively enrol patients and collect data. Therefore, as the pressure eased after the devastating 3^rd^ wave, the authors of this paper decided to collect data retrospectively within the time period of 01.03.2020–28.02.2021, encompassing the first surge in Spring and second wave in Autumn 2020. Participating centres were all related to the major SepsEast collaborators^7,9^ within Central and Eastern Europe—see list of participating centres in Table 1. The primary outcome of our study was all-cause in hospital mortality defined as a death during ICU stay or death occurring after transfer from ICU to the ward during the same hospitalization, and there was no censoring nor missing data of the main (primary) outcome.

**Table 1:** Participating centres.

Country—Centre	No of ICU patients	Percentage of the dataset (%)
**CROATIA**	**286**	**13**
University Hospital Rijeka	286	13
**CZECHIA**	**583**	**27**
University Hospital Plzen	583	27
**HUNGARY**	**269**	**13**
Flór Ferenc Hospital County Pest	112	5
University of Pécs, School of Medicine	157	7
**POLAND**	**115**	**5**
Poznań Medical University Hospital	66	3
Central Clinical Hospital of the Ministry of Interior and Administration, Warsaw	49	2
**SLOVAKIA**	**491**	**23**
University Hospital Nitra	178	8
University Hospital Nové Zámky	166	8
University Hospital Banska Bystrica	147	7
**SLOVENIA**	**395**	**18**
General Hospital Celje	226	11
General Hospital Murska Sobota	169	8
**Overall**	**2139**	100

### Patients

All consecutive adult patients admitted to the ICU due to COVID-19 pneumonia within the dedicated time period were found eligible. Patients admitted with severe acute respiratory failure due to other reasons than coronavirus 2 (SARS-CoV-2), but in whom SARS-CoV-2 screening proved positive on hospital or ICU admission, were excluded.

### Data collection

The following groups of parameters were searched for within hospital databases or patient records (Supplementary Table 1): baseline demographic parameters, comorbidities, time describing parameters (i.e. symptom onset, date of first proved SARS-CoV-2 positivity, admission and discharge/death dates), parameters of organ support (i.e. mode and length of ventilator support, other vital organ supports) and treatment (corticosteroids, anti-viral and disease modifying drugs, anticoagulation), ICU stay related complications (i.e. deep-vein thrombosis, pulmonary embolism, barotrauma), laboratory parameters on ICU admission (i.e. leukocyte, lymphocyte count, C-reactive protein, procalcitonin level, PaO_2_/FiO_2_ ratio). In patients who died in the ICU the most probable cause of death was identified by using the methodology of a recent study by Contou et al.^10^, and orders to either withhold or withdraw treatment were also screened. The parameters necessary for the main outcome analysis were available in all patients. However, because of the retrospective nature of the study not all secondary parameters were available for each patient, hence in the case of missing data, the individual patients were left out of the dedicated secondary analysis. We also circulated a questionnaire among all participating centres, asking them what had affected an unfavourable outcome the most in their opinion (Supplementary Table 2).

### Statistics

In order to minimize bias all centres that expressed voluntary interest in the study were included. Available data were summarised using descriptive statistics. For categorical values the counts and proportions (%) are depicted. For continuous variables the number of values (n) and depending on data distribution median, mean, interquartile range, were tabulated. Normality of data was tested by the Kolmogorov–Smirnov test. Mortality (understood as fatality rate) with 95% confidence intervals (95% CI) was calculated for participating centres, gender, age, comorbidity, and pandemic waves. An exploratory analysis was performed to investigate differences between survivors and non-survivors wherever appropriate. Further subgroup analysis of patients receiving invasive ventilation and those without invasive ventilator support was also conducted. Mann–Whitney, ANOVA on Ranks and chi-square tests were used respectively. All data were handled anonymously, MS Excel 2016 and MedCals software were used for all statistical analyses.

### Ethics approval and consent to participate

This study was conducted in accordance with the amended Declaration of Helsinki. The original, prospective study protocol was approved by the Hungarian National Research and Ethics Committee (IV/3971-3/2020/EKU) in May 2020 (Nemzeti Népegészségügyi Központ IV/3971-3/2020/EKU, 20.05.2020), but this study did not include any patients. The protocol of the retrospective data collection was first approved by the Ethical Committee University Hospital Pilsen and Faculty of Medicine in Pilsen, Charles University, reference number: 198/2021, then in the other centres:

- Clinical Hospital Center Rijeka: Ethics Committee, No: 2170-29-02/1-20-2.
- University of Pécs: Clinical Center Ethics Committee, No: KK/864-1/2021.
- Hospitals Celje and Musrka Sobota: Slovenia National Medical Ethics Committee No: 0120-168/2021/7.
- Krakow and Poznan: Bioethical Committee of the Jagiellonian University in Krakow, No: 1072.6120.105.2020.
- Nové Zamky: Etická komisia FNsP Nové Zámky Slovenská 11/A, Nové Zámky 94034.
- Fór Ferenc Hospital, Kistarcsa: Pest Megyei Flór Ferenc Kórház Intézeti Kutatási Etikai Bizottság (Institutional Research Ethics Committe, Flór Ferenc Hospital), No: ALT/3556-1/2021.
- University Hospital Nitra: Etická komisia Fakutna nemocnica Nitra.
- University Hospital Bansky Bystrica: Fakultná nemocnica F.D. Roosevelta, Banská Bystrica.

Because of its retrospective nature and handling of anonymised data, no patient approvals or consents were deemed necessary and were waived by the above listed ethics committees.

## Results

**Figure 1:**
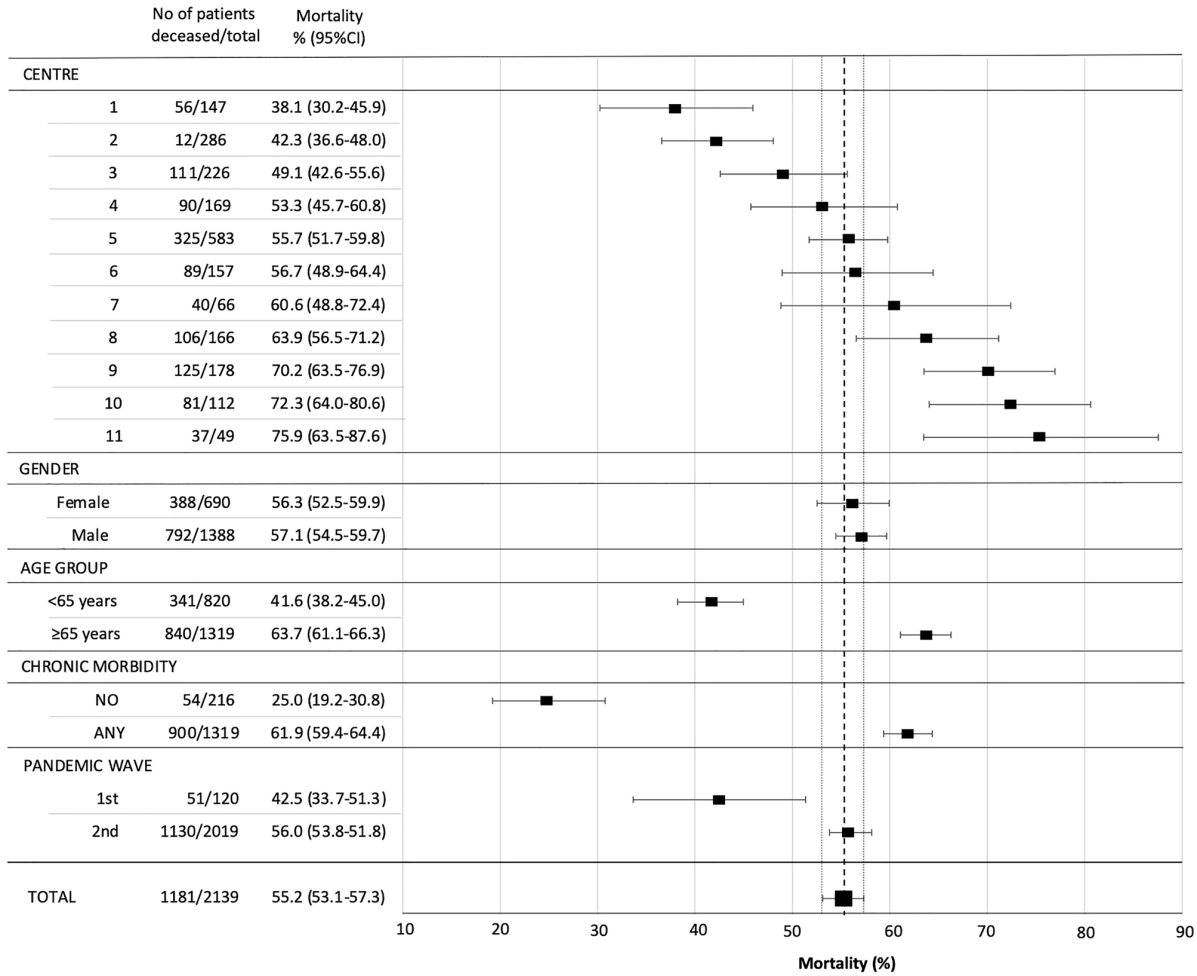
Hospital mortality in selected subgroups as compared to the overall cohort. Mortality is expressed in percentages and depicted as squares. Corresponding 95% confidence intervals (95% CI) are given in parentheses and shown as error bars. Dashed and dotted vertical lines represent mortality and boundaries of 95% CI in the overall cohort, respectively.

Over the study period 2139 patients were included from six Central-Eastern European countries and 11 centres (Table 1). Out of these, 958 survived and 1181 (55%) died in the ICU. Mortality was comparable among females (54%) and males (55%) (Fig. 1). Demographics and other important baseline characteristics for the whole cohort, and survivors and non-survivors are depicted separately in Table 2.

**Table 2:** Summary of demographics, complications and treatment characteristics.

	Overall N = 2139	Survivors N = 958	Non-survivors N = 1181	p-value
Age	68 (60–75)	65 (55–72)	70 (64–77)	< 0.0001
Female	690 (33%)	302 (34%)	388 (33%)	NS
BMI	30.1 (27.0–34.7)	30.7 (27.3–34.9)	30.0 (26.8–34.6)	NS
Onset of symptoms before ICU admission (days)	6 (2–9)	6 (3–9)	5 (2–8)	< 0.0001
ICU pre-admission hospital length of stay (days)	1 (0–4)	2 (0–4)	1 (1–5)	0.023
ICU length of stay (days)	9 (5–16)	10.5 (6–18)	8 (4–15)	< 0.0001
Organ support free total length of stay (days)	1 (0–4)	3 (1–7)	1 (0–3)	< 0.0001
**Comorbidities**	N = 1656	N = 475	N = 1181	
Without comorbidities	230 (12%)	162 (23%)	68 (6%)	< 0.0001
Diabetes mellitus	626 (38%)	165 (35%)	461 (39%)	NS
Arterial hypertension	1201 (73%)	475 (64%)	895 (74%)	NS
Chronic heart disease	575 (35%)	114 (24%)	461 (39%)	NS
Chronic respiratory disease	297 (18%)	80 (17%)	217 (18%)	NS
Immunocompromised (incl. dialysis, malignancy)	450 (27%)	91 (19%)	359 (30%)	NS
CPR before ICU admission	32 (2%)	7 (1%)	25 (2%)	NS
**Organ support**	N = 1687	N = 733	N = 954	
Only HFNC	317 (19%)	192 (26%)	125 (13%)	< 0.0001
Only NIV	129 (8%)	64 (9%)	65 (7%)	NS
Invasive ventilation w/o ECMO	1061 (63%)	357 (49%)	704 (74%)	NS
ECMO	54 (3%)	22 (3%)	32 (3%)	NS
Vasopressor therapy	1093 (65%)	365 (50%)	728 (76%)	< 0.0001
Inotropic support	200 (12%)	38 (5%)	162 (17%)	< 0.0001
RRT	205 (12%)	54 (7%)	151 (16%)	< 0.0001
**ICU complications**	N = 1656	N = 475	N = 1181	
Pulmonary embolism	106 (6%)	27 (6%)	79 (7%)	NS
HAP/VAP	444 (27%)	148 (31%)	296 (25%)	NS
Barotrauma	32 (2%)	5 (1%)	27 (2%)	NS
CPR	255 (15%)	7 (1%)	248 (21%)	NS
**Specific treatments**	N = 1744	N = 721	N = 1023	
Corticosteroids (any dose)	1520 (87%)	626 (87%)	894 (84%)	NS
Standard dose	1017 (58%)	424 (59%)	593 (58%)	NS
Higher dose	503 (29%)	202 (28%)	301 (29%)	NS
DVT prophylaxis	540 (31%)	218 (30%)	322 (31%)	NS
Anticoagulation (heparin or high-dose LMWH)	1128 (65%)	486 (67%)	642 (63%)	NS
Anti-platelets (chronic or new medication)	240 (14%)	93 (13%)	146 (14%)	NS
Antivirals (any of the following)	407 (23%)	196 (27%)	211 (21%)	NS
Remdesivir	264 (15%)	145 (20%)	119 (12%)	NS
Lopinavir/ritornavir	15 (1%)	8 (1%)	7 (1%)	NS
Favirapivir	178 (10%)	68 (9%)	110 (11%)	NS

Data are presented as median (25–75th percentile) and absolute number (percentage). For statistical analysis Mann–Whitney U test or Chi square tests were used where appropriate, NS—non-significant.

*BMI* body mass index, *CPR* cardio-pulmonary resuscitation, *DVT* deep vein thrombosis, *ECMO* extracorporeal membrane oxygenation, *HAP* healthcare-associated pneumonia, *HFNC* high-flow nasal cannula, *ICU* intensive care unit, *LMWH* low molecular weight heparin, *NIV* non-invasive ventilation, *RRT* renal replacement therapy, *VAP* ventilator-associated pneumonia.

### In hospital mortality

Although hospital mortality was the main (primary) outcome, out of the 1181 patients who died only 53 (4%) died outside the ICU—mostly on standard wards and/or long-term facilities. The median time to death after ICU discharge in these patients was 6 (IQR 3–14) days. Mortality (fatality rate) for the investigated domains is summarised in Fig. 1. There were substantial differences between the centres regarding risk of dying. Compared to the mortality in the overall cohort, patients had a significantly lower risk in two centres and a significantly higher risk for mortality in three centres.

There was a strong association with age (< 65 vs > 65 years) and the presence of any comorbidity vs. no comorbidity with increased risk of death (Fig. 1). Mortality was also substantially lower during the first (1–17 inclusion study weeks) compared to the second wave (study week 18–52).

**Figure 2:**
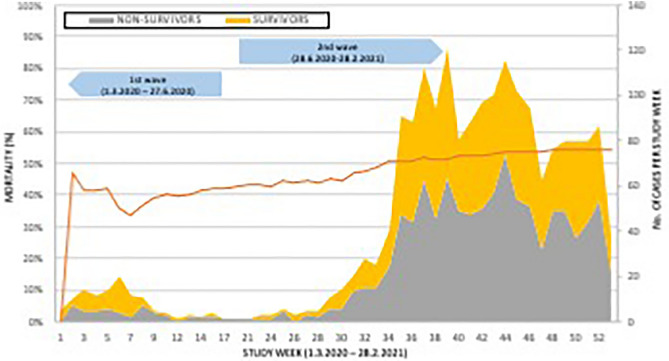
Distribution of survivors and non-survivors, and mortality during the study period. Absolute numbers of surviving (yellow) and non-surviving (grey) patients (right sided Y-axis) based on the inclusion study week (X-axis) are shown. Orange line represents mortality calculated per study week (left sided Y-axis). Arrows depict the limits of 1st and 2nd wave of COVID-19 pandemic.

Patient numbers increased dramatically during the second wave but there was also a significant increase in mortality over time (Fig. 2). During the first wave mortality in the overall cohort was 43% and increased to 56% during the second wave (p = 0.004). Mortality also increased significantly with age in both sexes (Supplementary Fig. 1).

**Figure 3:**
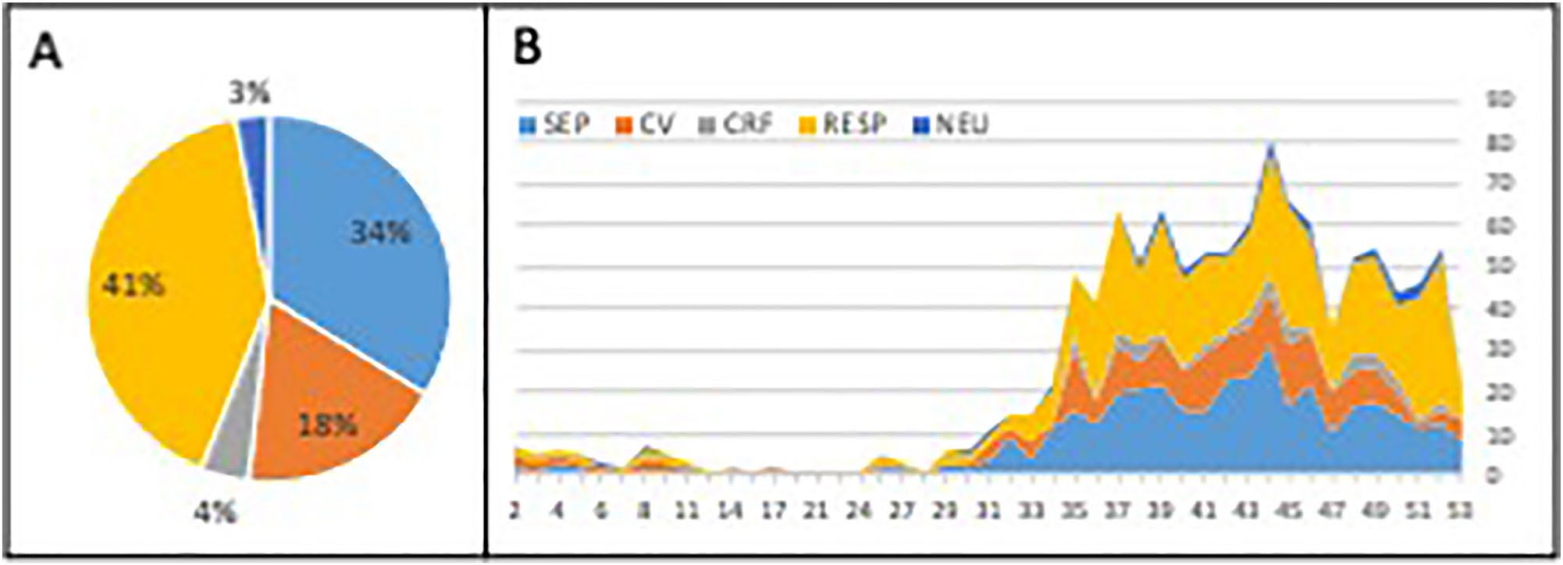
Major cause of death. Pie plot with overall distribution (**A**) and time-based evolution during the study (**B**; X-axis—study inclusion week, Y-axis—absolute number of patients) is presented for the following causes of death: sepsis and multi-organ failure (SEP, blue), cardiovascular failure (CV, orange), cardio-respiratory failure (CRF, grey), respiratory failure (RESP, yellow), neurological cause (NEU, dark blue).

The major cause of death was identified as respiratory failure followed by sepsis with multiorgan failure, cardiovascular failure, irreversible neurological damage and cardio-respiratory failure (Fig. 3A). There were no major differences in these causes between the 1st and 2nd waves (Fig. 3B).

Decisions on any form of treatment limitation were made in 35% of non-survivors, in whom treatment was withheld in 8% and withdrawn in 7%. There were no major differences over time or between the 1st and 2nd waves.

During the first day of hospitalization 191 patients died (16% of non-survivors). Out of these, treatment was withheld in 45 (24%) and withdrawn in 6 (3%) cases. The major causes of death among these patients were respiratory failure in 71 cases (37%), cardiovascular failure in 50 patients (26%) and sepsis with multiorgan failure in 40 patients. (21%). In 32 patients (2%) cardiopulmonary resuscitation was performed prior to or during the admission period (Table 1 section Comorbidities), but 7 (22%) of these survived to hospital discharge.

### Survivors vs. non-survivors

Survivors were significantly younger compared to non-survivors (Table 2). They also stayed a median of 1 day longer at home (from the onset of symptoms) and in hospital before warranting ICU admission. Their overall ICU stay was also longer, and median number of organ-support free days was three times lower than in non-survivors.

There was also a significant difference between survivors and non-survivors in terms of admission data (Table 3). Non-survivors were admitted in a significantly worse condition as indicated by higher sequential organ failure assessment (SOFA), acute physiology and chronic health evaluation (APACHE) II scores, serum lactate and inflammatory biomarkers (C-reactive protein, procalcitonin, interleukin-6 and ferritin), D-dimer levels, and lower PaO_2_/FiO_2_ ratio.

**Table 3:** Baseline parameters on ICU admission.

	Overall		Survivors		Non-survivors		p-value
	N	Median (IQR)	N	Median (IQR)	N	Median (IQR)	
SOFA	515	7 (4–10)	156	4 (2–8)	359	8 (5–11)	< 0.0001
APACHE II	551	17 (12–25)	187	13 (10–19)	364	21 (14–27)	< 0.0001
PaO_2_/FiO_2_ (mmHg)	1352	97 (66–150)	527	123 (80–200)	825	84 (62–124)	< 0.0001
Lymphocyte count (10^9^/L)	1286	0.46 (0.16–0.95)	424	0.70 (0.46–1.86)	862	0.32 (0.07–0.75)	< 0.0001
CRP (mg/L)	1711	118 (62–190)	706	104 (54–173)	1005	128 (70–205)	< 0.0001
PCT (ng/mL)	1490	0.39 (0.18–1.14)	501	0.30 (0.12–1.00)	989	0.41 (0.20–1.31)	< 0.0001
IL-6 (pg/mL)	448	68 (23–144)	155	45 (15–106)	293	88 (28–191)	< 0.0001
Ferritin (ųg/L)	939	1081 (580–2000)	351	797 (418–1542)	588	1311 (741–2030)	< 0.0001
D-dimers (mg/L)	1226	2.65 (1.22–9.00)	373	2.12 (1.13–7.32)	853	3.06 (1.26–10.19)	0.0083
Serum lactate (mmol/L)	1308	1.8 (1.3–2.9)	400	1.3 (1.0–1.8)	908	2.2 (1.5–3.5)	< 0.0001

Data are presented as median (25–75th percentile). For statistical analysis Mann–Whitney U test was used.

*IQR* interquartile range, *SOFA* Sequential Organ Failure Assessment, *APACHE II* Acute Physiology and Chronic Health Evaluation II score, *PaO*_*2*_*/FiO*_*2*_ Horowitz oxygenation index, *CRP* C-reactive protein, *PCT* procalcitonin, *IL-6* interleukin-6.

### Organ support

**Figure 4:**
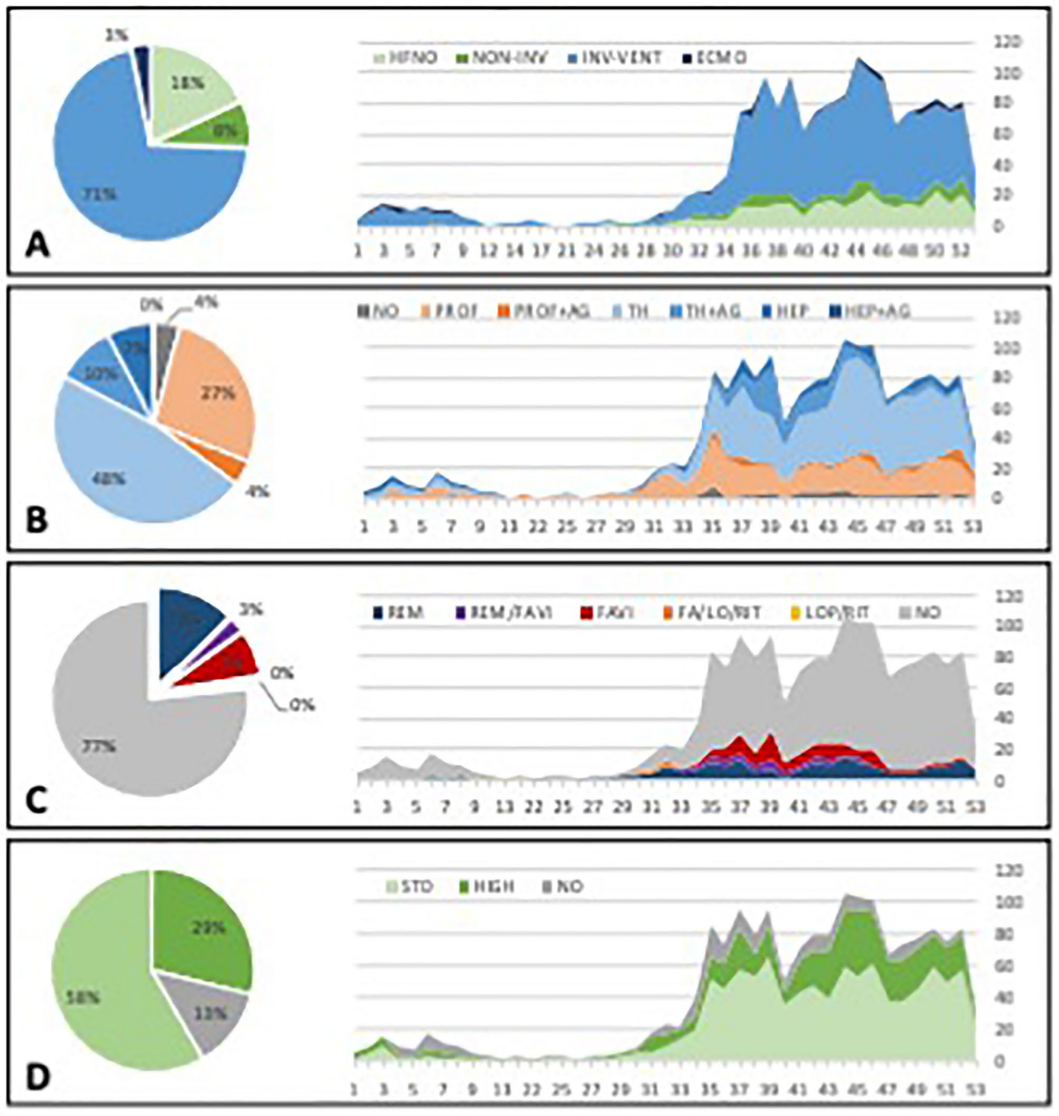
Disease specific treatment modalities. Each treatment modality is displayed as a pie plot for overall distribution and time-based evolution during the study (X-axis—study inclusion week, Y-axis—absolute number of patients). Respiratory support (**A**): high-flow nasal oxygen (HFNO, light green); non-invasive ventilation (NON-INV, dark green); invasive mechanical ventilation (INV-VENT, light blue); extracorporeal oxygenation (ECMO, dark blue). Anticoagulation and anti-aggregants (**B**): no anti-thrombotics (NO; grey); prophylactic low-molecular weight heparin (PROF, light orange); prophylactic low-molecular weight heparin + anti-aggregants (PROF + AG, dark orange); therapeutic low-molecular weight heparin (TH, very light blue); therapeutic low-molecular weight heparin + anti-aggregants (TH + AG, light blue); heparin anticoagulation (HEP, dark blue); heparin anticoagulation + anti-aggregants (HEP + AG—very dark blue). Antivirals (**C**): no antivirals (NO, grey); remdesivir (REM, dark blue); favirapivir (FAVI, red); lopinavir-ritornavir combination (LOP/RIT, yellow) and their potential combinations. Corticosteroids (**D**): without steroids (NO, grey); standard dose of dexamethasone 6-8 mg/day equivalents (STD, light green); any higher dose (HIGH, dark green).

Out of the whole cohort 54 patients (3%) received extracorporeal membrane oxygenation (ECMO) of whom 59% died (Table 2, Fig. 4A). Invasive mechanical ventilation (without ECMO) was necessary in 1061 patients (63%) (Fig. 4A) with an overall mortality of 66% (Table 2, Supplementary Fig. 2). In the remaining 572 (34%) patients without invasive ventilation mortality was significantly lower (38%, p < 0.0001; Supplementary Fig. 2). In 405 patients (24%) non-invasive ventilation was commenced and in 276 cases (68%) it had to be escalated to invasive mechanical ventilation. Mortality was 50% in 129 (32%) patients receiving non-invasive ventilation as their highest level of ventilatory support.

High-flow nasal oxygen (HFNO) therapy was provided in 913 patients (54%). This proved to be sufficient in 317 (35%) of these patients (i.e., HFNO only), and the remainder required further escalation of their ventilatory support. Mortality in those receiving HFNO only was 39%. In 126 patients, conventional oxygen therapy was not escalated, and the mortality in this group was 22%.

Interestingly 434 patients received invasive mechanical ventilation as the first choice of support without previous HFNO/non-invasive attempts. Mortality in this group was the highest (70%) and 109 (25%) of them died within the first day of hospitalization.

Vasopressors were prescribed in 1093 (65%) patients and inotropic support was used in 200 (12%) patients. Any form of renal replacement therapy was required in 205 (12%) patients. Need for each of these supports significantly increased patients’ mortality (Table 2).

### Disease specific treatments

There was marked evolution of divergent treatment approaches over the study period. The time evolution and proportion of patients receiving various antithrombotic prophylaxis (panel B), antiviral medication (panel C) and corticosteroids (panel D) is depicted in detail in Fig. 4.

### Subjective assessment of factors affecting unfavourable outcomes

**Figure 5:**
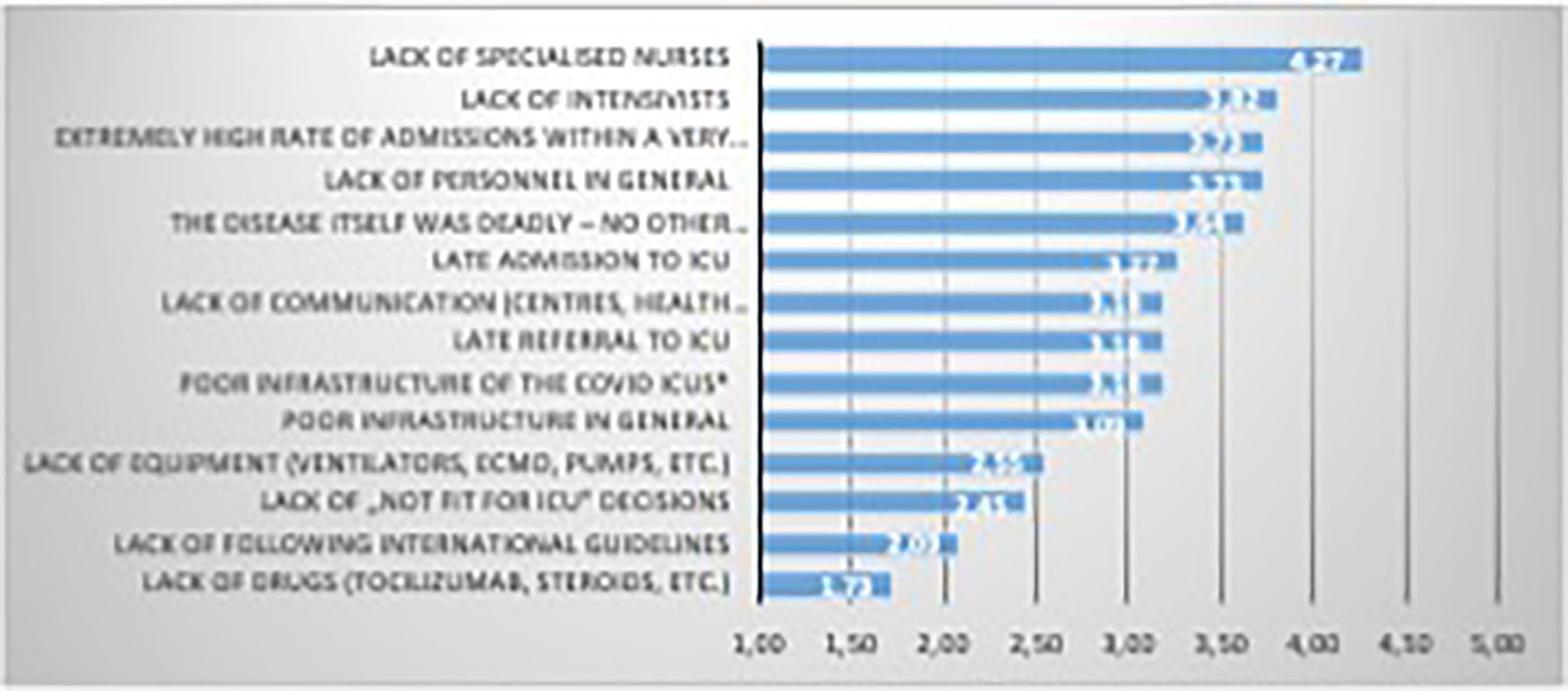
Factors subjectively associated with unfavourable outcome. Results of the survey among participating centres are presented for each factor as weighted average of the following rating: not important (1), slightly important (2), important (3), fairly important (4), very important (5).

Lack of specialised nurses was indicated as the most important factor affecting mortality by most participating centres. Amongst the 5 factors with the highest rankings, this was followed by: extremely high rate of admissions within a very short period, lack of intensivists, lack of personnel in general and deadly character of the disease (COVID-19) (Fig. 5).

## Discussion

The SEARCH-COVID-19 study analysed the characteristics and outcomes of 2139 ICU patients during the 1st and 2nd waves of the COVID-19 pandemic. To the best of our knowledge, this is the largest dataset from Central-Eastern European countries on this patient population to date. This multicentre, international study with a comprehensive dataset on COVID-19 patients treated on the ICUs in six Central-Eastern European countries revealed that both overall mortality and especially those receiving invasive mechanical ventilation had substantially higher mortality compared to that reported in previous studies from Western European countries.

The 2 years of the COVID-19 pandemic represent an unprecedented period in medicine, challenging health care systems all around the globe and provoking research activity the like of which the medical community has never seen before. Intensive care medicine has played a crucial part in the fight against the devastating effects of the virus. Despite all efforts, more than 5.6 million people have died around world to date due to the corona virus affecting every single country on the planet^11^. The number of patients infected, admissions to hospital and ICU, and mortality have varied over time and by country to country. In a recent article Islam and colleagues showed that COVID-19 resulted in a huge number of excess deaths exceeding the reported actual COVID-19 deaths and suggested that assessment of the full impact of the pandemic on mortality should include both the direct effect of the pandemic and the indirect influence on deaths from other causes associated with the disruption to health services or wider economic and social changes^8^. Even though national governments have reported the number of deaths from COVID-19 daily, scientific publications from Central-Eastern Europe remain scarce. This has also been shown by a recent meta-analysis in which all papers reporting case fatality rates were analysed, and although Europe contributed thousands of patients from several countries, no suitable study was found during the systematic search that included countries from Central-Eastern Europe^12^.

### Patient characteristics and management

Regarding age, patients in our cohort admitted to ICU belonged primarily to the elderly population (> 65 years of age) with higher prevalence of males than females. These and also the patients’ body mass index were similar to that of reported by other studies^3,5,12^. Only 12% of patients were free of any comorbidities, which is substantially lower than reported by one Italian cohort (32%)^4^, but similar to that of found in another study (22%)^5^. Patients were admitted to ICU 5–6 days after the onset of symptoms and stayed on the ward 1–2 days in general. This is again similar to that observed in other studies^13,14^, but 2 days shorter than reported in non-survivors in a French single centre study^10^.

The general condition of the patients on admission as indicated by APACHE II scores were similar to that of reported in the Intensive Care National Audit & Research Centre (ICNARC) database^3^. Admission median SOFA score of the non-survivors in our study was 8, which is substantially higher than that reported in a recent single centre study of 73 deceased patients (median 4, IQR 3–8)^10^. These data suggest that our population was slightly sicker but there are no obvious differences that have been shown to be associated with worse outcomes in COVID-19 patients.

We also analysed the cause of death, which was in accord with that reported in other studies, with refractory respiratory failure and sepsis accounting for 75% of all deaths. This was found to be the same in a single centre French study^10^.

The median duration of stay in the ICU in our study was 9 days (10.5 for survivors, 9 for non-survivors) which was longer than in the ICNARC dataset (5 days for both survivors and non-survivors)^3^, but similar to that of reported by others^10,15^. Regarding treatment modalities our data suggest that patients in general were treated more-or-less according to international recommendations.

These data suggest that based on the overall characteristics our patient population was not that different to those included elsewhere in Europe. Furthermore, it is highly likely that patients received similar treatment modalities including ventilatory support, medication and ECMO.

### Mortality

The most important results of our study are the higher overall mortality especially those needing mechanical ventilation in our region compared to most of the reports coming from the Western European countries. External validity of our results is compromised due to the relatively small sample size and limited number of participating ICUs. Therefore, we cannot conclude that our results are generalizable for the whole region. However, as there are no other large sample size studies published on this topic to contradict our results and taking into account the epidemiological data published on international official websites^6^, we consider the message of our data as an alarming signal that should be taken seriously and investigated thoroughly in the future.

The odds ratio for mortality showed substantial differences in the participating centres, but this heterogeneity is a common feature all around the world as indicated for example by the meta-analysis by Lim et al.^12^. Despite the low number of patients, ICU mortality during the 1st wave was around 40%, which increased during the 2nd wave to 56%. As compared to international data, in one of the very first reports on 1590 patients from Grasselli et al., 88% of patients were mechanically ventilated and overall mortality was 25%^4^. The “COVID-19 Italian ICU Network” reported that out of 1260 patients treated during the 1st wave, 79% underwent invasive mechanical ventilation and that mortality among intubated patients was 38.5%^5^. In 551 patients from 7 ICUs located in The Euroregio Meuse-Rhine mortality during the 1st wave of COVID-19 differed significantly between participating countries and was 22%, 42% and 44% in Belgium, The Netherlands and Germany, respectively (but only 53% of patients in Belgian ICUs were invasively ventilated)^16^. In the United Kingdom, according to the ICNARC reports, during the 1st wave, overall mortality was 50.7% which reduced to 35.2% in the report released a year later on the 26 February 2021^3^.

Data from other countries are less positive. In Germany despite the drop in ICU admissions during the second wave of the pandemic, the mortality of mechanically ventilated patients remained unchanged and also above 50%^17^. According to the COVID-19 SEMICYUC Working Group (from Andora, Ireland and Spain) mortality was lower, but without significant differences between the waves (31.7% versus 28.8%)^18^.

Although a multicentre international study in 2625 patients also reported reduced survival rate in the 2nd wave (30 days: 1st wave 43% vs 2nd wave 50%, and 90 days: 1st wave 49% vs 2nd wave 60%), this study only included the elderly population aged 70 years and older^15^. In this study out of the 14 countries, there was only 1 included from Central-Eastern Europe (Poland), who contributed 102 patients from 12 centres, hence comparisons between their results and ours are difficult to make. Nevertheless, this data also supports the finding in our study, that the 1st wave in Central-Eastern Europe was less severe as compared to the West as, from the 12 Polish centres, only 12 patients were included during the 1st and 90 in the 2nd wave.

In our dataset, 66% patients were mechanically ventilated which is substantially less than the 88% in the previously mentioned Italian study, but the mortality showed a dramatic difference of 66% in our study versus 25% in theirs^4^. The same conclusion can be drawn when we compare our results to that of reported by ICNARC on 6501 invasively ventilated patients where mortality was 46.8% during the 2nd wave^3^. Furthermore, overall mortality in a meta-analysis on invasively ventilated patients all around the world was 45% (95% confidence interval 39–52%), which is again lower than in our cohort^12^.

Regarding in hospital mortality of patients receiving only non-invasive respiratory support, it was 39% for HFNO, 50% for non-invasive ventilation and 22% for conventional oxygen therapy. These results are worse when compared to the most recent results of the RECOVERY-RS trial, which revealed that hospital mortality occurred in 21.2%, 19.8% and 22.4% in patients on HFNO, continuous positive airway pressure (CPAP) and conventional oxygen therapy, respectively^19^.

Unfortunately, we have limited published data from Central-Eastern European ICUs^20–23^ to compare our data to, but the current results on mortality match with those published on the national and international websites, suggesting that mortality of COVID-19 patients in the six countries included in the current analysis was somewhat higher than in our Western European counterparts^6^.

Undoubtedly, we cannot present data that could explain this potential difference in mortality between the two parts of Europe (i.e.: Central-East vs West). One cannot exclude that the indication and potential delay in commencing the invasive ventilator support may be one of the critical points as to why patients in the 2nd wave of our study did worse than others. Our patients’ overall median APACHE II score was 17 and it was higher than in Belgian and German ICUs but similar to Dutch centres participating in The Euroregio Meuse-Rhine study^16^. Mortality of patients admitted with a similar APACHE II score (17+) in the ICNARC database also had similar 28-day mortality to that of ours of around 57% as reported on 26.02.2021^3^. Furthermore, the median PaO_2_/FiO_2_ on admission was 99 mmHg in our study, that is lower than reported in some other studies with better outcomes^3,4^. These may suggest that our patients, despite the crude similarity in demographics and patient characteristics were still sicker.

Furthermore, there are some well-known circumstances that may have played an important contribution if this difference truly exists. On the one hand, the “one way traffic” of health care personnel from countries of the former socialist block of Europe (i.e.: Central-Eastern Europe) including the six countries participating in the current study has been going on for decades. Although this exodus of the Central-Eastern European work force to the West is well known and acknowledged, it has never been audited, researched, and most importantly never been published in scientific journals. Therefore, the subjective assessment of the authors of the situation, that the most important factor of unfavourable outcome in COVID-19 critically ill patients might be related to lack of personnel, should be taken seriously. Although a recent publication coming from Australia-New Zealand found no association between patient-to-intensivist ratio and hospital mortality^24^, these results may not be applicable for the eastern part of Europe for reasons pointed out earlier and will also be discussed later. Even in the editorial for the same article the authors clearly emphasize the potential importance of the strain on the critical care workforce^25^ that is also supported by recent publications^26,27^. Adding these issues to the lack of trained personnel could indeed have a major impact on outcomes.

On the other hand, it has also been well documented that the Gross Domestic Product (GDP) related spending on health care is substantially less in Central-Eastern Europe then in Western Europe (in Supplementary Table 3: Source 6 and 7). This could also have a profound effect on the observed exodus from East to the West, which may be an important potential factor of worse outcomes than that reported from Western European countries.

Last but not least, structured training and overall motivation of junior doctors and other health care workers, especially specialised nurses, should also be reviewed and improved^7^.

Finally, to confirm or contradict the validity of our results, nationwide and internationally, well structured, transparent, trustworthy audits, registries and studies are needed, ideally supported by governmental funding. The goal should be to develop a system and structure, which is similar to that of those developed in Western Europe and in the United Kingdom^3,5^.

### Strengths and limitations

Although this is the most comprehensive and largest dataset ever published from Central-Eastern Europe on COVID-19 patients treated on the ICU, it has several limitations. The most important is that our dataset cannot be considered as representative data for the whole region, not even for these six countries, which limits its external validity. Nevertheless, it is important to note that Central-Eastern European clinical research was unable to compete in the publication “race” neither before, nor during the pandemic. The contribution of this part of Europe to the unprecedented number of scientific papers published from all over the world remains negligible. In our view this is a system failure that is also supported by the fact that we could not include a single patient in the SEARCH study prospectively, simply because of the inadequate staffing levels on the ICU who were overwhelmed, and the almost complete lack of research dedicated personnel. This leads to the other important limitation, which is the retrospective nature of the study, resulting in reduced number of data on COVID-19 related admissions and outcomes during this period, organ support related parameters which we could not collect retrospectively, nor the patient-to-nurse/physician ratio, or specialised nurse-to-patient ratio.

## Conclusion

This is the largest and most comprehensive COVID-19 dataset from Central-Eastern European ICUs suggesting the potential high mortality rate observed especially in those receiving invasive mechanical ventilation. There is still a plausible difference in quality of health care and research output between the East and West that has not changed for almost two decades since our joining the European Union. Our results render the need of a paradigm change in Central-Eastern Europe to establish high quality, structured data collection and to improve research facilities and output, all contributing to better patient outcomes in Central-Eastern Europe.

## Supplementary Information

Supplementary Information.

## Data Availability

Data may be available for research purposes on request. For this purpose please contact the first or the corresponding authors: JB, MJ, AS, ZM.

## References

[1] Remuzzi A, Remuzzi G (2020). COVID-19 and Italy: What next?. Lancet.

[2] World Health Organization (2021) COVID-19 Weekly Epidemiological Update. https://www.who.int/publications/m/item/weekly-epidemiological-update-on-covid-19. (Supplementary Table 3, S1). Accessed 27 April 2021.

[3] Intensive Care National Audit and Research Centre (ICNARC) (2022) Case Mix Programme database reports. https://www.icnarc.org/Our-Audit/Audits/Cmp/Reports (Supplementary Table 3, S2, S5). Accessed 31 Jan 2022.

[4] Grasselli G, Zangrillo A, Zanella A, Antonelli M, Cabrini L, Castelli A, Cereda D, Coluccello A, Foti G, Fumagalli R, Iotti G, Latronico N, Lorini L, Merler S, Natalini G, Piatti A, Ranieri MV, Scandroglio AM, Storti E, Cecconi M, Pesenti A, COVID-Lombardy ICU Network (2020). Baseline characteristics and outcomes of 1591 patients infected with SARS-CoV-2 admitted to ICUs of the Lombardy Region, Italy. JAMA.

[5] Zanella A, Florio G, Antonelli M, Bellani G, Berselli A, Bove T, Cabrini L, Carlesso E, Castelli GP, Cecconi M, Citerio G, Coloretti I, Corti D, Dalla Corte F, De Robertis E, Foti G, Fumagalli R, Girardis M, Giudici R, Guiotto L, Langer T, Mirabella L, Pasero D, Protti A, Ranieri MV, Rona R, Scudeller L, Severgnini P, Spadaro S, Stocchetti N, Vigano M, Pesenti A, Grasselli G, COVID-Italian ICU Network (2021). Time course of risk factors associated with mortality of 1260 critically ill patients with COVID-19 admitted to 24 Italian intensive care units. Intensive Care Med..

[6] Our World In Data. (2022) Coronavirus Pandemic (COVID-19). https://ourworldindata.org/coronavirus (accessed 31.01.2022). (Supplementary Table 3, S3) Accessed 31 Jan 2022.

[7] Molnar Z (2017). SepsEast: Bridging between East and West. J. Crit. Care.

[8] Islam N, Shkolnikov VM, Acosta RJ, Klimkin I, Kawachi I, Irizarry RA, Alicandro G, Khunti K, Yates T, Jdanov DA, White M, Lewington S, Lacey B (2021). Excess deaths associated with covid-19 pandemic in 2020: Age and sex disaggregated time series analysis in 29 high income countries. BMJ.

[9] Lainscak M, Sustic A, Benes J, Czuczwar M, Jankovic R, Kirov M, Kula R, Kusza K, Podbregar M, Sandesc D, Bedreag O, Szuldrzynski K, Zahorec R, Hegyi P, Molnar Z (2020). SepsEast and COVID-19: Time to make a difference. Signa Vitae..

[10] Contou D, Cally R, Sarfati F, Desaint P, Fraisse M, Plantefeve G (2021). Causes and timing of death in critically ill COVID-19 patients. Crit. Care..

[11] Center for Systems Science and Engineering (CSSE) at Johns Hopkins University (JHU). COVID-19 Dashboard. https://www.arcgis.com/apps/dashboards (Supplementary Table 3, S4). Accessed 31 Jan 2022.

[12] Lim ZJ, Subramaniam A, Ponnapa Reddy M, Blecher G, Kadam U, Afroz A, Billah B, Ashwin S, Kubicki M, Bilotta F, Curtis JR, Rubulotta F (2021). Case fatality rates for patients with COVID-19 requiring invasive mechanical ventilation. A meta-analysis. Am. J. Respir. Crit. Care Med..

[13] Boelle PY, Delory T, Maynadier X, Janssen C, Piarroux R, Pichenot M, Lemaire X, Baclet N, Weyrich P, Melliez H, Meybeck A, Lanoix JP, Robineau O (2020). Trajectories of hospitalization in COVID-19 patients: An observational study in France. J. Clin. Med..

[14] Patel BV, Haar S, Handslip R, Auepanwiriyakul C, Lee TM, Patel S, Harston JA, Hosking-Jervis F, Kelly D, Sanderson B, Borgatta B, Tatham K, Welters I, Camporota L, Gordon AC, Komorowski M, Antcliffe D, Prowle JR, Puthucheary Z, Faisal AA, United Kingdom COVID-ICU National Service Evaluation (2021). Natural history, trajectory, and management of mechanically ventilated COVID-19 patients in the United Kingdom. Intensive Care Med..

[15] Jung C, Fjolner J, Bruno RR, Wernly B, Artigas A, Bollen Pinto B, Schefold JC, Wolff G, Kelm M, Beil M, Sviri S, van Heerden PV, Szczeklik W, Czuczwar M, Joannidis M, Oeyen S, Zafeiridis T, Andersen FH, Moreno R, Leaver S, Boumendil A, De Lange DW, Guidet B, Flaatten H, COVIP Study Group (2021). Differences in mortality in critically ill elderly patients during the second COVID-19 surge in Europe. Crit. Care..

[16] Mesotten A, Meijs DAM, van Busses BCT, Stessel B, Mehagnoul-Schipper J, Hana A, Scheeren CIE, Strauch U, Ghossein-Doda C, Buhre WFFA, Bickenbach J, Vander Laenen M, Marx G, van der Horst ICC (2022). COVID Data Platform (CoDaP) Investigators: Differences and similarities among COVID-19 patients terated in seven ICUs in three countries within one region: An observational cohort study. Crit. Care Med..

[17] Karagiannidis C, Windisch W, McAuley DF, Welte T, Busse R (2021). Major differences in ICU admissions during the first and second COVID-19 wave in Germany. Lancet Respir. Med..

[18] Carbonell R, Urgeles S, Rodriguez A, Bodi M, Martin-Loeches I, Sole-Violan J, Diaz E, Gomez J, Trefler S, Vallverdu M, Murcia J, Albaya A, Loza A, Socias L, Ballesteros JC, Papiol E, Vina L, Sancho S, Nieto M, Lorente MDC, Badallo O, Fraile V, Armestar F, Estella A, Sanchez L, Sancho I, Margarit A, Moreno G, COVID-Semicyuc Working Group (2021). Mortality comparison between the first and second/third waves among 3795 critical COVID-19 patients with pneumonia admitted to the ICU: A multicentre retrospective cohort study. Lancet Reg Health Eur..

[19] Perkins GD, Ji C, Connolly BA, Couper K, Lall R, Baillie JK, Bradley JM, Dark P, Dave C, De Soyza A, Dennis AV, Devrell A, Fairbairn S, Ghani H, Gorman EA, Green CA, Hart N, Hee SW, Kimbley Z, Madathil S, McGowan N, Messer B, Naisbitt J, Norman C, Parekh D, Parkin EM, Patel J, Regan SE, Ross C, Rostron AJ, Saim M, Simonds AK, Skilton E, Stallard N, Steiner M, Vancheeswaran R, Yeung J, McAuley DF (2022). Effect of noninvasive respiratory strategies on intubation or mortality among patients with acute hypoxemic respiratory failure and COVID-19: The RECOVERY-RS randomized clinical trial. JAMA.

[20] Czapla M, Juarez-Vela R, Gea-Caballero V, Zielinski S, Zielinska M (2021). The association between nutritional status and in-hospital mortality of COVID-19 in critically-ill patients in the ICU. Nutrients.

[21] Gjurasin B, Santini M, Krajinovic V, Papic N, Atelj A, Kotarski V, Krznaric J, Vargovic M, Kutlesa M (2021). A retrospective comparison between influenza and COVID-19-associated ARDS in a Croatian tertiary care center. Wien Klin Wochenschr..

[22] Kokoszka-Bargiel I, Cyprys P, Rutkowska K, Madowicz J, Knapik P (2020). Intensive care unit admissions during the first 3 months of the COVID-19 pandemic in Poland: A Single-Center, Cross-Sectional Study. Med. Sci. Monit..

[23] Moiseev S, Avdeev S, Brovko M, Bulanov N, Tao E, Fomin V (2021). Outcomes of intensive care unit patients with COVID-19: A nationwide analysis in Russia. Anaesthesia.

[24] Gershengorn HB, Pilcher DV, Litton E, Anstey M, Garland A, Wunsch H (2022). Association of patient-to-intensivist ratio with hospital mortality in Australia and New Zealand. Intensive Care Med..

[25] Kerlin MP, Caruso P (2022). Towards evidence-based staffing: The promise and pitfalls of patient-to-intensivist ratios. Intensive Care Med..

[26] Wahlster S, Sharma M, Lewis AK, Patel PV, Hartog CS, Jannotta G, Blissitt P, Kross EK, Kassebaum NJ, Greer DM, Curtis JR, Creutzfeldt CJ (2021). The coronavirus disease 2019 pandemic's effect on critical care resources and health-care providers: A global survey. Chest.

[27] Kerlin MP, Silvestri JA, Klaiman T, Gutsche JT, Jablonski J, Mikkelsen ME (2022). Critical care clinician wellness during the COVID-19 pandemic: A longitudinal analysis. Ann. Am. Thorac. Soc..

