# Supplementary material for: SepsEast Registry indicates high mortality associated with COVID-19 caused acute respiratory failure in Central-Eastern European intensive care units
Source: Sci Rep. 2022 Sep 1;12:14906. doi: 10.1038/s41598-022-18991-2 (PMC9436166; doi:10.1038/s41598-022-18991-2)
Supplement: Supplementary file 1 — Supplementary Information. [file 41598_2022_18991_MOESM1_ESM.docx]

**Supplementary material**

**Supplementary Figure S1.** Patient distribution and mortality by gender and age groups

**
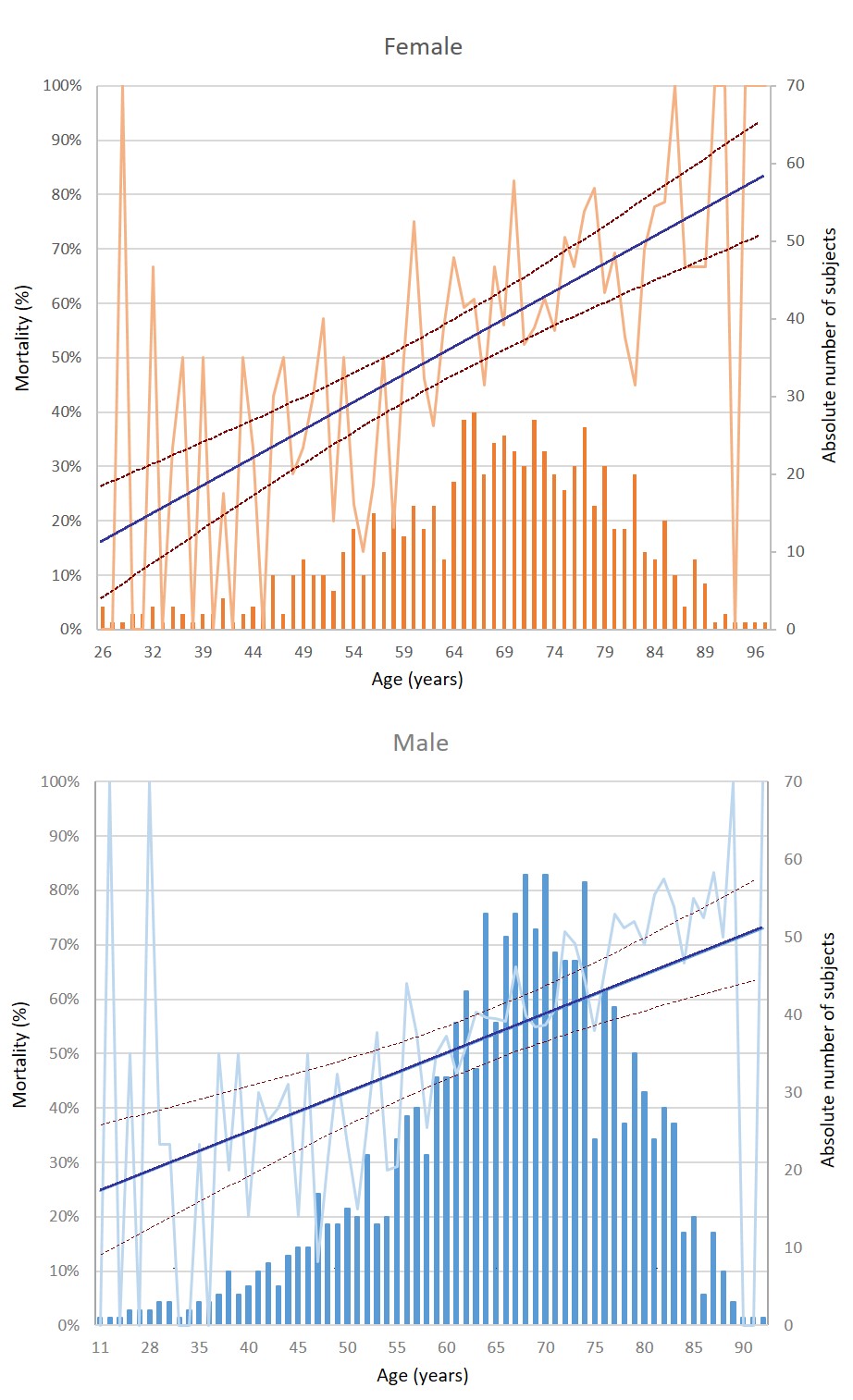
**

Columns depict absolute number of patients (right-sided Y-axis) by 1-year of age group (X-axis). Calculated mortality (left-sided Y-axis, light blue/orange line) with regression line and 95% confidence intervals (solid blue and dashed red lines) is shown.

**Supplementary Figure S2.** Patient distribution based on ventilator support.

Absolute numbers (blue bars, left-sided Y-axis), and mortality (orange bars, right-sided Y-axis) are shown for the following groups: patients with known organ support without ECMO patients (Overall), patients with need for invasive mechanical ventilation (Mechanically ventilated), patients without invasive ventilator support, i.e.: non-invasive ventilation, high-flow nasal oxygen, conventional oxygen therapy (W/o invasive ventilation).

**Supplementary Table 1.** **Screened variables**

|  | Centre ID* |
| --- | --- |
|  | Patient ID* |
| DATES | Disease onset* |
|  | Proved SARS CoV2 positivity (PCR or AG)* |
|  | Hospital admission* |
|  | ICU admission* |
| DEMOGRAPHICS | Age* |
|  | Gender (M/F)* |
|  | BMI |
|  | Heigh (only if BMI is not known) |
|  | Weight (only if BMI is not known) |
| PATIENT HISTORY (mark only positive with "Y" or "X") | Obesity |
|  | Diabetes mellitus (I or II) |
|  | Arterial hypertension |
|  | Immunocompromised (transplant, long term medication etc.) |
|  | Chronic kidney disease (incl. Dialysis) |
|  | Chronic respiratory disease |
|  | Chronic heart disease |
|  | Peripheral arterial disease |
|  | Cancer/hemato-oncological treatment |
|  | CPR before admission |
|  | No of chronic medication |
|  | SARS CoV2 vaccinated |
|  | COVID re-infection |
| ORGAN SUPPORT (mark only positive with "Y" or "X"/or give DATE) | Invasive ventilation (Y/N or date of onset) |
|  | Length of invasive ventilation (days) |
|  | Extracorporeal support (ECMO/ECCO2R) (Y/N or date of onset) |
|  | Length of extracorporeal support (ECMO/ECCO2R) (days) |
|  | High flow oxygen |
|  | Noninvasive ventilation |
|  | Vasopressors |
|  | Inotropes/chronotropes |
|  | Dialysis |
| COMPLICATIONS (mark only positive with "Y" or "X") | Venous thrombosis |
|  | Pulmonary embolism |
|  | Hospital acquired pneumonia |
|  | Other nosocomial infection (not pneumonia) |
|  | Hemophagocytic lymphohistiocytosis |
|  | CPR (including before as a cause for admission) |
|  | Other………… |
| DISCHARGE AND DEATH  (cause of death: describe in plain words into the relevant group collumn) | Date of ICU discharge* |
|  | Where (standard, long-term facility, home)* |
|  | Date of death* |
|  | WITHHOLD treatment order – no escalation |
|  | WITHDRAWN treatment order - palliative care |
|  | Respiratory failure |
|  | Cardiovascular collapse incl. arrhythmias |
|  | Sepsis/infection/multiorgan failure |
|  | Neurological (both bleeding and ischemic) |
|  | Other ………….. |
|  |  |
| ICU ADMISSION SCORING AND LABORATORY ( | SOFA |
|  | PaO2/FiO2 (spontaneous/ on respiratory support) |
|  | APACHE II |
|  | Lymphocyte count (minimal) |
|  | CRP |
|  | PCT |
|  | IL6 |
|  | Ferritin |
|  | D-Dimer |
|  | Lactate |
| ICU SPECIFIC TREATMENTS | Corticosteroids (standard dosing) |
|  | Corticosteroids (higher dosing) |
|  | Tocilizumab |
|  | Remdesivir |
|  | Other antivirotic agent (name) |
|  | Monoclonal antibody (name) |
|  | Covalescent plasma |
|  | Isoprinosine |
|  | Ivermectine |
|  | Hydroxychlorochine |
|  | Vitamine D |
|  | Vitamine C + Thiamine |
|  | Heparin |
|  | LMWH prophylactic dose |
|  | LMWH full therapeutic dose |
|  | Other anitcoagulants / fibrinolytics / antiaggregants (descibe) |
|  | Sel- proning during HFO/NIV |
|  | Proning after intubation |
|  | Muscle relaxants (prolonged use to facilitate ventilation) |
|  | Cytokine removal (i.e. CytoSorb) |

Mandatory variables are indicated by asterisks; collection of the remaining variables was based on availability.ICU admission data are relevant for all lab/scoring variables

**Supplementary Table 2. Survey questionnaire**

| To which extent the following factors affected the unfavourable outcome in your centre based on your opinion | NOT important | Slightly important | Important | Fairly important | Very important |
| --- | --- | --- | --- | --- | --- |
| *The disease itself was deadly  – no other confounders* |  |  |  |  |  |
| *Lack of personnel in general* |  |  |  |  |  |
| *Lack of specialised nurses* |  |  |  |  |  |
| *Lack of intensivists* |  |  |  |  |  |
| *Poor infrastrurcture in general* |  |  |  |  |  |
| *Poor infrastructure of the COVID ICUs** |  |  |  |  |  |
| *Late referral to ICU* |  |  |  |  |  |
| *Late admission to ICU* |  |  |  |  |  |
| *Extremely high rate of admissions within a very short period of time* |  |  |  |  |  |
| *Lack of „not fit for ICU” decisions* |  |  |  |  |  |
| *Lack of equipment (ventilators, ECMO, pumps, etc.)* |  |  |  |  |  |
| *Lack of drugs (tocilizumab, steroids, etc.)* |  |  |  |  |  |
| *Lack of following international guidelines* |  |  |  |  |  |
| *Lack of communication (centres, health authorities, etc)* |  |  |  |  |  |
| *Other comment….* |  |  |  |  |  |
| The questionnaire was circulated among participating centers using Surveymonkey.com web application. The principal investigator was responsible for filling in the questionnaire.  * These are ICUs outside the regular critical care area, usually located in wards temporarily allocated for caring of critically ill patients with COVID-19 | | | | | |

**Supplementary Table 3.** **Supplementary references**

| Reference no. | Website address |
| --- | --- |
| S1 | World Health Organization. Coronavirus disease (COVID-19) situation reports. Geneva, Switzerland: World Health Organization;2020 [accessed 2020 Jul 30]. Available from: https://www. who.int/emergencies/diseases/novel-coronavirus-2019/situation- reports |
| S2 | ICNARC (Intensive Care National Audit and Research Centre) Case Mix Programme database report 24 April 2020. Accessed from https://[www.icnarc.org](http://www.icnarc.org) |
| S3 | <https://ourworldindata.org/coronavirus> [assessed 2022 Jan 1]. |
| S4 | <https://www.arcgis.com/apps/dashboards/bda7594740fd40299423467b48e9ecf6> [accessed 2022 Jan 13] |
| S5 | ICNARC (Intensive Care National Audit and Research Centre) Case Mix Programme database report 21 February 2021. Accessed from https://[www.icnarc.org](http://www.icnarc.org) |
| S6 | https://data.worldbank.org/indicator/SH.XPD.CHEX.GD.ZS?name_desc=false [accessed 2022 Jan 24] |
| S7 | <https://apps.who.int/nha/database/ViewData/Indicators/en> [accessed 2022 Jan 24] |

S, source
